# Electrodeposited Ultrathin TiO2 Blocking Layers for Efficient Perovskite Solar Cells

**DOI:** 10.1038/srep16098

**Published:** 2015-11-03

**Authors:** Tzu-Sen Su, Tsung-Yu Hsieh, Cheng-You Hong, Tzu-Chien Wei

**Affiliations:** 1 Department of Chemical Engineering, National Tsing-Hua University, Taiwan

## Abstract

In this study, the electrodeposition (ED) of ultrathin, compact TiO_2_ blocking layers (BLs) on fluorine-doped tin oxide (FTO) glass for perovskite solar cells (PSCs) is evaluated. This bottom-up method allows for controlling the morphology and thickness of TiO_2_ films by simply manipulating deposition conditions. Compared with BLs produced using the spin-coating (SC) method, BLs produced using ED exhibit satisfactory surface coverage, even with a film thickness of 29 nm. Evidence from cyclic voltammetry shows that an ED BL suppresses interfacial recombination more profoundly than an SC BL does, consequently improving the photovoltaic properties of the PSC significantly. A PSC equipped with an ED TiO_2_ BL having a 13.6% power conversion efficiency is demonstrated.

Since the organo-lead halide perovskite light absorber was reported in 2009^1^, the perovskite solar cell (PSC) has developed into a promising category in the photovoltaic territory. Since the first PSC with a double-digit power conversion efficiency (PCE) having an analogous structure to solid-state dye-sensitised solar cells was reported in 2012^2^, the device architecture of the PSC has been broadened to include several format, such as extremely thin absorber cells^2^, meso-superstructured solar cells^3^, and planar heterojunction cells^4^,^5^. Planar junction PSCs with an extremely high PCE (over 20%) have been demonstrated and certificated by the National Renewable Energy Laboratory, USA (http://www.nrel.gov/). Within only five years, PSCs have become a viable competitor to conventional silicon-based photovoltaics. In a typical PSC, when incident light is absorbed by organo-lead halide perovskite, photo-generated electron-hole pairs are separated and then injected into n-type and p-type semiconductors, respectively. Since carriers are transported via different routes and collected at two terminals, careful control of the various materials and interfaces, including the blocking layer (BL)^5^,^6^,^7^, the perovskite layer^8^,^9^,^10^, and the hole transport layer^11^,^12^,^13^, is critical to achieving a high PCE.

Among the various materials and interfaces, the BL refers to the individual layer between the transparent conductive substrate and the light absorbing layer. The BL plays a crucial part in the PSC because it prevents carriers from directly contacting the conductive substrate and thereby shunting the device. Such a charge recombination has been proven to lower charge collection efficiency, and leads to lowering the short circuit current (J_SC_) as well as the fill factor (FF) on the current–voltage (IV) characteristic curve of a PSC^6^. TiO_2_ is the most frequent BL material used^14^, although zinc oxide^15^ and cesium carbonate^16^ are also high-performing materials. Spin coating (SC) and spray pyrolysis (SP) are two representative solution processes used to form BLs^4^,^17^,^18^,^19^, although atomic layer deposition (ALD)^6^, thermal oxidation^20^, and TiCl_4_ chemical bath deposition^21^ have also been reported in the literature. SC involves dipping a few solution containing a dilute titanium precursor onto a vacuum-suctioned fluorine-doped tin oxide (FTO) substrate, then spinning the shaft and baking the substrate sequentially to form the film. SP involves using an atomiser to spray a titanium precursor onto a heated substrate; the precursor droplets thermally decompose simultaneously to form the film. It is generally acknowledged that the quality of BLs produced through either SC or SP is highly sensitive to process parameters, and thus the PCE can vary substantially even when applying the same BL process. Ideally, a BL should be pinhole-free and compact at very thin thicknesses to avoid additional resistive losses. In practice, producing perfectly covered films on such a small scale continues to be challenging^22^. Both SC and SP are top–down strategies: the TiO_2_ precursor is randomly driven by auxiliary forces from the bulk area to the substrate surface; these methods thus lack the controllability to form a naturally continuous film. Satisfactory coverage is usually accomplished by increasing the film thickness, which simultaneously increases the internal resistance of the device.

ED is a bottom-up methodology that provides a contour-chasing capability because the electrochemical reaction occurs on the electrode surface. In contrast to other bottom-up methods such as ALD, ED is a simple, cost-effective, and scalable method to obtain a continuous and uniform surface coating without the need for a high temperature treatment or vacuum environment. More important, compared with ALD, the deposition rate of ED is one order of magnitude higher than ALD, which is advantageous when considering mass production of PSC in the future. Moreover, by manipulating the parameters of ED, both film thickness and morphology can be easily controlled. Kavan *et al.* reported that TiO_2_ can be formed through the fast hydrolysis of Ti^3+^ in an acid solution followed by a galvanic reaction, resulting in a perfectly covered TiO_2_ layer^23^. Wu *et al.* applied this method not only to deposit a TiO_2_ BL on the FTO surface^24^,^25^ but also to modify the connection of TiO_2_ nanoparticles^26^ for dye-sensitised solar cells. In this article, we apply ED from an aqueous TiCl_3_ solution to prepare a BL on an FTO glass for TiO_2_-scaffold type PSCs for the first time. Characterisations from field-emission scanning electron microscopy (SEM), secondary ion mass spectrometry (SIMS), and cyclic voltammetry (CV) show that the ED BL is thinner and denser; it thus exhibits an enhanced blocking performance compared with an SC BL. As a result, PSCs equipped with ED BLs outperform those equipped with SC BLs by more than 3% in PCE.

## Results & Discussion

**Figure 1:**
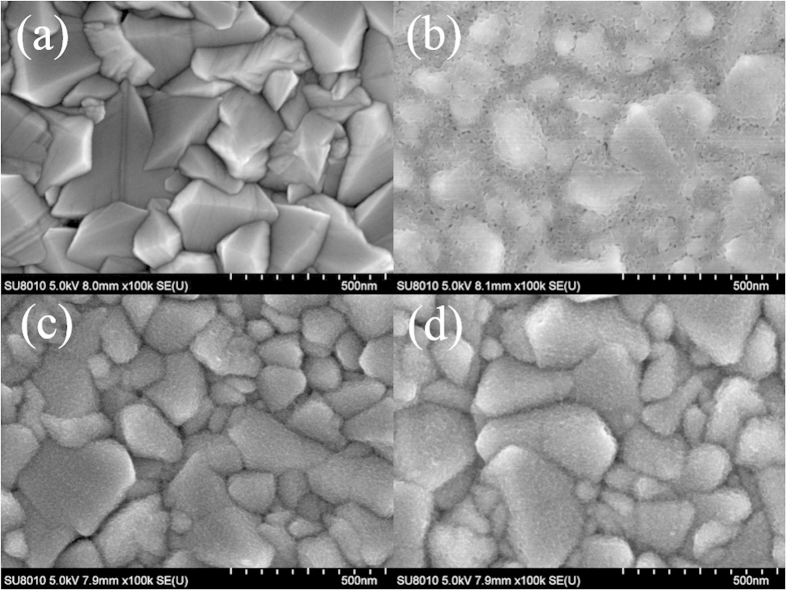
SEM topographies of (**a**) bare FTO, (**b**) SC-TiO_2_ on FTO, (**c**) ED-BL-20-500, and (**d**) ED-BL-20-500 after sintering.

First, the identity of ED TiO_2_ film was confirmed by X-ray diffractometer. To enhance the diffraction signal, we purposely electrodeposited a thick TiO_2_ film by extending deposition time to 4000 seconds. As shown in Fig. S1, diffraction peaks assigned for the anatase TiO_2_ (JCPDS card no.86-1155) appeared after annealing ED TiO_2_ at 450 °C for 30 minutes, confirming the chemical identity of ED TiO_2_ film. Figure 1 shows SEM topographies of FTO substrates with and without different BL coatings. As shown in Fig. 1a, bare FTO grains with irregular shapes ranging from tens to hundreds of nanometres in size were observed, leading to a rough surface. For SC BLs, Fig. 1b shows that the FTO grains were overlaid by a film composed of many visually observable nano-pinholes. The thickness of the SC BL on FTO glass is approximately 60 nm, and thus the FTO grains are blurred. Figure 1c is an image of the ED BL produced with a current density of 20 μA/cm^2^ for 500 seconds (denoted as ED-BL-20-500); FTO grains remain visually recognisable, but the sharpness of grain edges is slightly vague, implying that the ED BL film is extremely thin. Magnified images of Fig. 1c are provided in Fig. S2, in which a scale-like ED TiO_2_ film was grown on the FTO surface with no visually observable defects, indicating a satisfactory surface coverage. It has been reported that the structure of the BL may change because of the internal stress of the deposited film after the annealing process in subsequent scaffold layer preparations^27^. Consequently the ED-BL-20-500 sample was subjected to annealing at 500 °C for 30 minutes; Fig. 1d shows the resulting SEM image. As shown in Fig. 1d, the coverage of the ED BL remains unchanged and is visually well covered.

**Figure 2:**
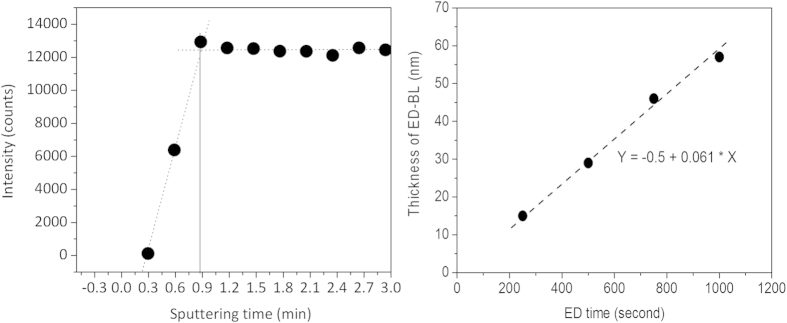
Time-of-flight secondary ion mass spectroscopy (TOF-SIMS) profile for the different coulomb densities of ED TiO2 blocking layers. The reference sputtering rate of SiO2 is 5.5 Å/s.

Figure S3 shows the cross-sectional view of SEM of ED BL. It revealed that the ED BL is very thin and continuous. However, as mentioned previously, the ED BL is too thin to identify its thickness by SEM. Commercial profile meters cannot be used because the roughness of the FTO is substantially larger than the thickness of the ED BL. We therefore applied SIMS to determine the thickness of the ED BL. Figure 2a is the SIMS depth profile of ED-BL-20-500. The thickness is determined as the signal of secondary tin ion is saturated, which means the ion beam has reached the valley of the FTO surface. From this, the ED BL thickness is estimated to be approximately 29.0 nm, which is approximately half the thickness of the SC BL. The relationship between film thickness and ED time shows that the deposition rate is approximately 0.061 nm/s, as depicted in Fig. 2b.

**Figure 3:**
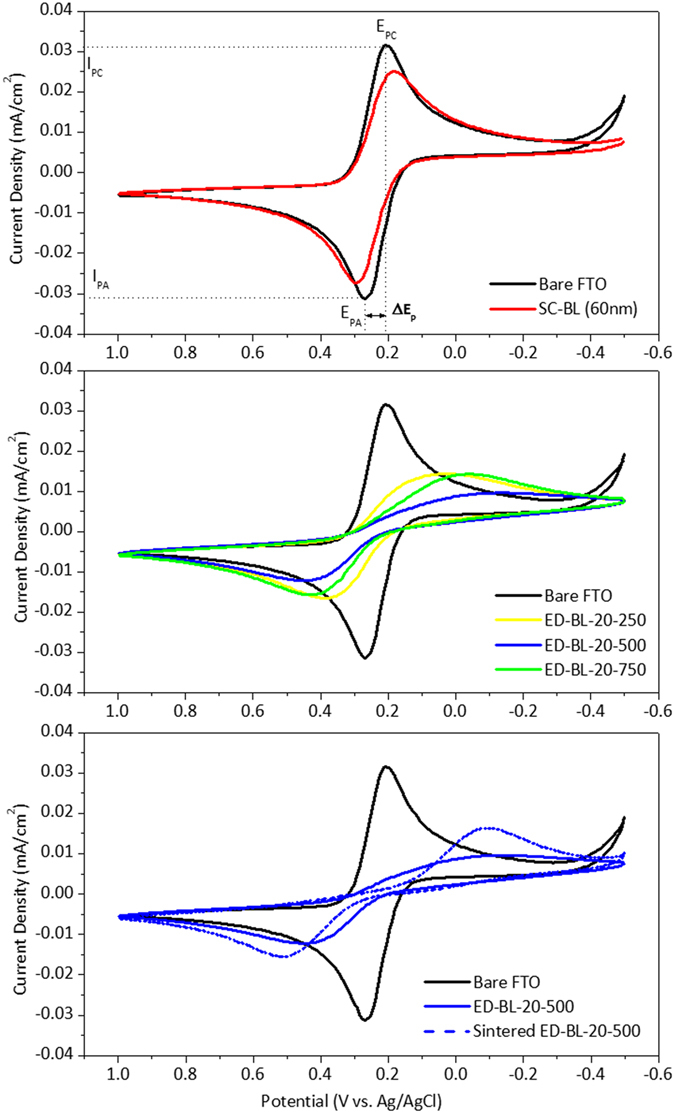
(**a**) CV waves of bare FTO and SC BL, (**b**) CV waves of ED BLs, and (**c**) CV waves of ED-BL-20-500 before and after post-sintering treatment.

CV was used to probe the blocking effect of the ED BL because it is an easy and efficient method^27^. An aqueous Fe(CN)_6_^3−/4−^ solution is typically used as the model redox system in a one-compartment, three-electrode electrochemical cell, in which FTO glass with or without a BL acts as the working electrode. By scanning CV on the working electrode in the aqueous Fe(CN)_6_^3−/4−^ electrolyte, the voltammogram representing the redox reaction between Fe(CN)_6_^3−^ and Fe(CN)_6_^4−^ is interpreted and used as the index of the blocking effect for a BL. Figure 3 shows the CV waves of bare and BL-coated FTO glass. The CV wave of bare FTO glass (black line in Fig. 3a) shows a Nernstian response in which the cathodic peak potential (E_PC_) and the anodic peak potential (E_PA_) are separated by 60.90 mV. This peak-to-peak separation (ΔE_P_) is reasonably deviated from the theoretical value of 56.00 mV for a one-electron reversible reaction because the uncompensated resistances of the solution, wiring, and electric contacts were not calculated. This result indicates that the redox reaction of Fe(CN)_6_^3−/4−^ ions is electrochemically reversible on bare FTO glass and thus no blocking effect exists. To further proof this electrochemical reversibility, multiple scan rates were also carried out and shown in Fig. S4. In addition, the peak current density, I_P_, is given by the Randles-Servcik equation:


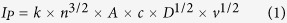


where k is a constant, n is the number of electrons transferred in the redox couple, A is the electrode area, D is the diffusion coefficient, and ν is the scan rate. The reaction of interest is the cathodic peak in the CV scan and its peak current density is proportional to the electrode area, A, when other parameters in reaction (1) remain unchanged. Because the FTO surface is coated with a BL, the electrode area available for reaction is reduced, leading I_P_ to decrease accordingly. Therefore, the ratio of cathodic I_P_ can be used as an index to express the surface coverage for a BL coating. In the case of an SC BL (red line in Fig. 3a), the ΔE_P_ and I_P(BL)_/I_P(FTO)_ are 110 mV and 0.795, respectively, indicating a blocking effect of only 20% peak current inhibition. As evidenced by SEM morphology (Fig. 1b), pinholes on the SC BL account for the result. The dependence of the blocking effect and ED BL thickness was evaluated using three ED BL samples prepared with an identical current density of 20 μA/cm^2^ but at different durations: 250, 500, and 750 seconds, respectively (denoted as ED-BL-20-250, ED-BL-20-500, and ED-BL-20-750, respectively). Shown in Fig. 3b and Table 1, the ΔE_P_ of ED-BL-20-250, ED-BL-20-500, and ED-BL-20-750 are 360.2, 598.4, and 465.4 mV, respectively. This indicates that the redox reaction between Fe(CN)_6_^3−/4−^ ions retards significantly and becomes semi reversible; moreover, the cathodic waves of the ED BL are deformed to a broad peak, implying that the ED BL is densely compact and that the electrons are hopping through the layer instead of having direct contact^28^,^29^. The I_P(BL)_/I_P(FTO)_ of ED-BL-20-500 (blue line) is 0.303, which is lowest among the three ED BLs; 500 seconds therefore appears to be an optimal depositing time in this preliminary study. ED-BL-20-250 (yellow line) may be too thin, allowing electrons to hop easily, rendering an I_P(BL)_/I_P(FTO)_ of 0.453. The I_P(BL)_/I_P(FTO)_ of ED-BL-20-750 is 0.451, a higher value than that of ED-BL-20-500; we speculate that because the ED film is sufficiently thick, it undergoes a structural deformation and recombination at the crevice of the thick ED BL. As indirect evidence, the CV peaks of ED-BL-20-750 (green line) appear Nernstian-like rather than sigmoidal with a steady-state current (as shown with ED-BL-20-500), implying the existence of small pinholes or defects in the ED-BL-20-750 sample.

When the ED-BL-20-500 sample was sintered, the CV wave (dotted blue line in Fig. 3c) changed significantly and two sharp, Nernstian-like peaks reappeared. This means structural defects were generated and thus more of the FTO surface area was naked during sintering, rendering an enlarged I_P(BL)_/I_P(FTO)_ of 0.518, as shown in Table 1. This phenomenon is attributable to volume shrinkage during the crystallisation of TiO_2_. Since sintering is a necessary step to forming TiO_2_ scaffolds on FTO glass, it is reasonable to conclude that the ED BL does not currently work under optimal conditions. This warrants further study. Nevertheless, compared with the SC BL, the sintered ED-BL-20-500 significantly outperforms in blocking the charge transfer between Fe(CN)_6_^3−/4−^ ions. Not only the recombination reaction become semi reversible because the ΔE_P_ of the CV wave in ED-BL-20-500 is significantly larger than that in the SC BL but also the surface coverage of the BL on the FTO is improved because the I_P(BL)_/I_P(FTO)_ of ED-BL-20-500 is 2.62 times smaller than that of the SC BL. Our CV analysis provides a prospective application of ED BLs in PSCs fabricated on a plastic substrate, in which high temperature sintering is prohibited; a room-temperature ED process seems favourable and promising.

**Figure 4:**
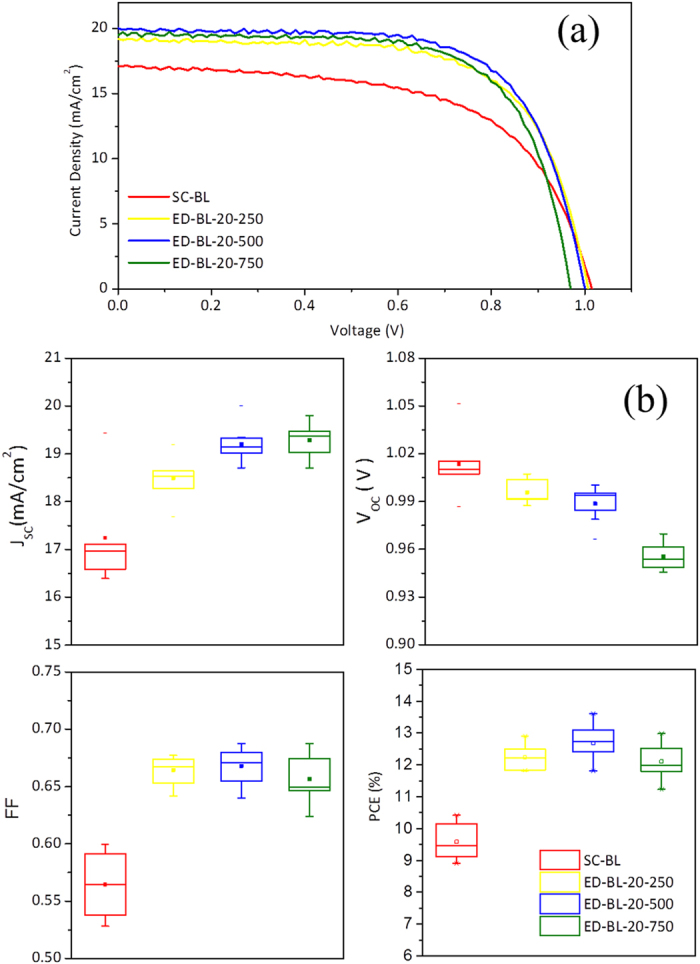
(**a**) IV-curves of best-performing PSC employed with different BL, (**b**) averaged IV parameters of PSC employed with different BL.

The photovoltaic performance of PSCs equipped with different BLs is demonstrated in Fig. 4. Figure 4a shows the IV characteristics of the best performing PSCs and Table 2 summarises their IV parameters. The PSC using an SC BL shows a J_SC_ of 17.11 mA/cm^2^, V_OC_ of 1015 mV, and FF of 0.60, yielding a PCE of 10.42%. The PSC using an ED-BL-20-500 delivers a J_SC_ of 20.01 mA/cm^2^, V_OC_ of 1000 mV, FF of 0.68, and an improved PCE of 13.60%. Fig. 4b is a statistical chart of IV parameters among six SC-BL-employed PSCs and ten ED-BL-employed PSCs; their averaged IV parameters are listed in Table 2. In both the best performing and average data, PCE improvements for ED BLs are attributable to J_SC_ and FF. The increase in J_SC_ is attributable to a suppressed recombination at the FTO surface, improving charge collection efficiency. The increase in FF is a combined result of thin and compact coatings on the FTO for ED BL, considerably minimising the impact of ohmic resistance.

In conclusion, we demonstrated that ED of TiO_2_ from an aqueous TiCl_3_ solution is an efficient method to fabricate ultrathin and compact BLs for PSCs. In this preliminary study, the film thickness growth rate was approximately 0.061 nm/s and the optimal ED condition was 20 μA/cm^2^ for 500 seconds. Our data revealed that sintering the as-prepared ED BL tended to induce structural defects that could not be examined in SEM but was detectable in CV scans, thereby reducing the blocking effect. Despite this, ED BLs still outperformed commonly used SC BLs in both the blocking effect and series resistance, leading to an improvement of J_SC_ and FF. Future investigations should optimise ED parameters such as the current density, coulomb density, and current profile (for instance, pulse current) to understand the relation between film morphology and the blocking effect and to further improve the photovoltaic performance of PSCs.

## Methods

### Fabrication of TiO_2_ blocking layer

FTO substrate (2.2 mm, 8 Ω/sq, NSG, Japan) was cleaned by commercial detergent (PK-LCG46, USA) and DI-water in sequence under ultrasonic bath for 20 minutes. TiO_2_ layer was anodically electrodeposited onto clean FTO substrate, which serves as the working electrode in a three-electrode compartment cell in a bath containing 0.25 M TiCl_3_ in water (20% in 3% HCl, Alfa Aesar) at room temperature. In this three-electrode system, an Ag/AgCl electrode filled with 3M NaCl solution and a 1 cm^2^ platinum foil were used as reference electrode and counter electrode, respectively. During entire deposition, the bath was kept at pH 2.5 by Na_2_CO_3_ and purged with nitrogen to prevent Ti^3+^ from oxidation. The experimental factors in this study are current density and depositing time (total passing coulomb density). The as-deposited ED-BL was rinsed by deionized water and dry in air. For comparison, SC-BL was also prepared by spin-coating an ethanol solution containing 0.2 M titanium isopropoxide (TTIP, 97%, Sigma-Aldrich) precursor and 0.2 M HCl 2000 rpm for 30 seconds, followed by a annealing treatment at 500 °C for 30 minutes in air.

### Fabrication of perovskite solar cell

Commercial TiO_2_ paste (30 nm, Dyesol, 30NRT) was diluted with pure ethanol at a ratio of 1/3.5 (w/w) and spin-coated at 4000 rpm for 30 seconds onto afore-prepared BL as mesoporous scaffold, followed by drying at 120 °C for 5 minutes and annealing at 500 °C for 30 minutes in sequence. The resultant film is then immersed into a chemical bath containing 40 mM TiCl_4_ under 70 °C for 30 minutes and followed by annealing again at 450 °C for 30 minutes. The fabrication of perovskite layer on BL-contained TiO_2_ scaffold was basically followed by published sequential deposition^9^. In brief, PbI_2_ (99.999%, Sigma-Aldrich) was dissolved in N,N-dimethylformamide (DMF) at a concentration of 1 M under stirring at 70 °C. The TiO_2_ scaffold was infiltrated by PbI_2_ by spin-coating (6000 rpm for 5 seconds) and followed by drying at 70 °C for 30 minutes. After cooling to room temperature, the substrates were immerged into a solution containing 0.1 M methylamine iodide (MAI, home-made) in 2-propanolfor 50 seconds, then rinsed with 2-propanol, dried by nitrogen flow and baked on 70 °C for 20 minutes. Hole transport material (HTM) was deposited by spin-coating a chlorobenzene solution containing 7.5wt% 2,2′,7,7′- tetrakis(*N*,*N*-di-*p*-methoxyphenylamine) -9,9-spirobifluorene (spiro-OMeTAD, Lumtec, Taiwan) at 3000 rpm for 30 seconds. In order to increase hole mobility of HTM layer, 20 mM bis(trifluoromethane)-sulfonimide lithium salt (Li-TFSI, Sigma-Aldrich) and 120 mM 4-tert- butylpyridine (TBP, 96%, Sigma-Aldrich) were added in above-mentioned spiro-OMeTAD solution. After storing the device at dry atmosphere overnight, a gold electrode was thermally evaporated onto HTM layer as the contact electrode. The entire fabrication process was carried out in a humidity-controlled room.

### Characterization and measurement

The topography of BL was scrutinized by FE-SEM (Hitachi SU-8010, Japan). The blocking effect of BL was investigated by CV provided by a computer-controlled potentialstat (Solartron SI 1286, UK) in a three-electrode system with Ag/AgCl reference electrode and 1 cm^2^ platinum counter electrode, respectively. In CV scan, the aqueous electrolyte containing 0.5 mM potassium hexacyanoferrate(II) trihydrate and 0.5 mM potassium hexacyano- ferrate(III) was utilized with 0.5M KCl as supporting electrolyte. All scan rate of CV measurements were performed at 50mV/s. The I-V characteristic of PSC was measured by a digital source meter (Keithley 2400, USA) under 1 Sun, AM1.5G illumination (Peccell Technologies, PEC-L15, Japan). A KG3 monocrystalline silicon photodiode (Oriel, USA) was used to calibrate the light intensity for better accuracy. A 0.088 cm^2^ photo-mask was attached to the front side of the PSC to precisely control the illuminating area.

## Additional Information

**How to cite this article**: Su, T.-S. *et al.* Electrodeposited Ultrathin TiO_2_ Blocking Layers for Efficient Perovskite Solar Cells. *Sci. Rep.* **5**, 16098; doi: 10.1038/srep16098 (2015).

## Supplementary Material

Supplementary Information

## Figures and Tables

**Table 1:** Summary of CV characteristics of different BLs prepared in this study (all potentials are versus to Ag/AgCl).

	Bare FTO	SC-BL 60 nm	ED-BL-20			
			250	500	750	500(sintered)
E_PC_ (mV)	208.2	184.4	32.1	−150.6	−34.8	−90.2
E_PA_ (mV)	269.2	294.4	392.3	447.8	430.6	513.8
ΔE_P_ (mV)	60.9	110.0	360.2	598.4	465.4	604.0
I_PC_ (μA/cm^2^)	31.56	25.10	14.30	9.58	14.24	16.36
I_PC(BL)_/I_PC(FTO)_	—	0.795	0.453	0.303	0.451	0.518

**Table 2:** Photovoltaic parameters of PSCs with different blocking layers.

Type of BL		J_SC_(mA/cm2)	V_OC_ (mV)	FF	PCE (%)
Best performing	ED-BL-20-250	19.20	1007	0.67	12.90
	ED-BL-20-500	20.01	1000	0.68	13.60
	ED-BL-20-750	19.47	970	0.69	12.98
	SC-BL	17.11	1015	0.60	10.42
Average	ED-BL-20-250	18.49	996	0.66	12.24
	ED-BL-20-500	19.20	989	0.67	12.69
	ED-BL-20-750	19.29	955	0.66	12.10
	SC-BL	17.24	1014	0.56	9.58
