# Supplementary material for: Electrodeposited Ultrathin TiO2 Blocking Layers for Efficient Perovskite Solar Cells
Source: Sci Rep. 2015 Nov 3;5:16098. doi: 10.1038/srep16098 (PMC4630649; doi:10.1038/srep16098)
Supplement: Supplementary Information [file srep16098-s1.pdf]

Supplementary Information

Electrodeposited Ultrathin TiO_2 Blocking Layers for Efficient Perovskite Solar Cells

Tzu-Sen Su‡, Tsung-Yu Hsieh‡, Cheng-You Hong, Tzi-Chien Wei*

Department of Chemical Engineering, National Tsing-Hua University, Taiwan

‡ The first two authors have contributed equally to this work

*Corresponding author: Tzu-Chien Wei

Tel: +886-3-5715131 ext.33669

E-mail: tcwei@mx.nthu.edu.tw

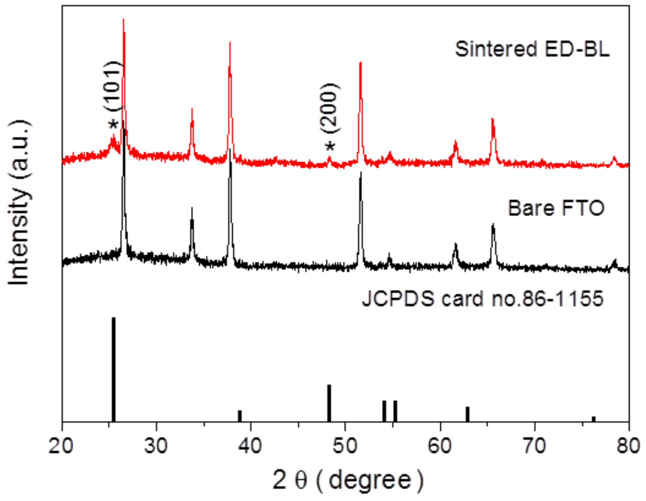

Figure S1. XRD patterns of the bare FTO substrate and titanium oxide coated FTO substrate after annealing at 450°C for 1h.

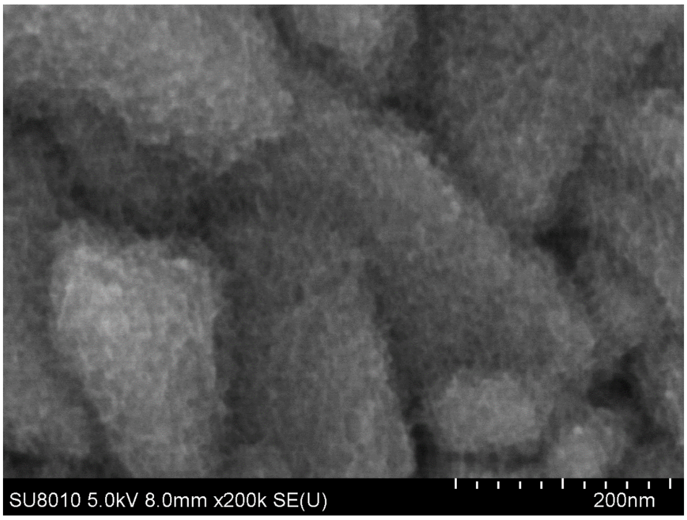

Figure S2: High magnification images of ED-BL-20-500.

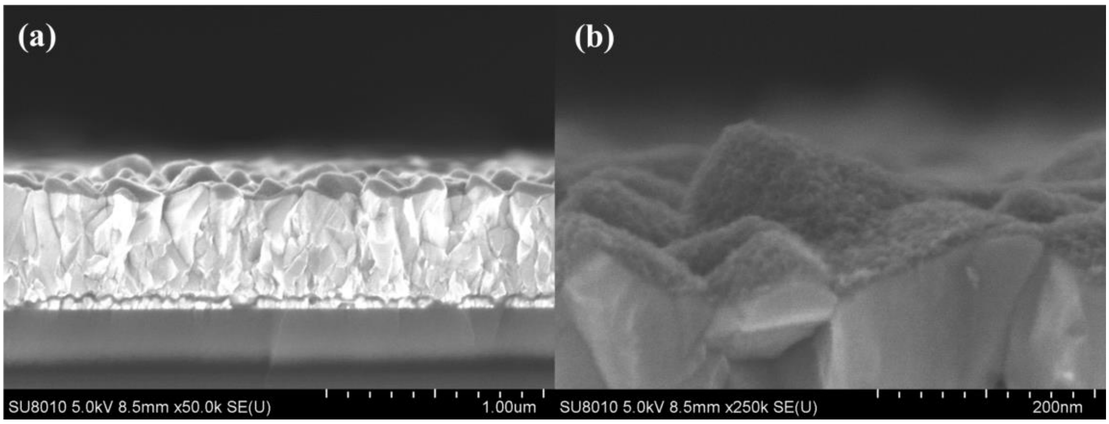

Figure S3. Cross-sectional SEM images of ED-BL-20-500 in (a)50k, (b)250k magnifications.

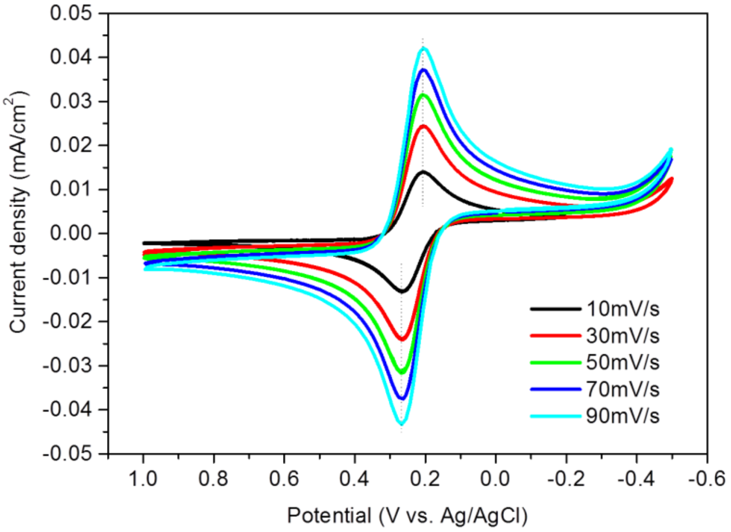

Figure S4. CV waves of bare FTO with multiple scan rates.
